# Social Inference May Guide Early Lexical Learning

**DOI:** 10.3389/fpsyg.2021.645247

**Published:** 2021-05-21

**Authors:** Alayo Tripp, Naomi H. Feldman, William J. Idsardi

**Affiliations:** ^1^Department of Speech-Language-Hearing Sciences, University of Minnesota, Minneapolis, MN, United States; ^2^Department of Linguistics, University of Maryland, College Park, MD, United States; ^3^Institute for Advanced Computer Studies, University of Maryland, College Park, MD, United States

**Keywords:** testimony, language acquisition, infant development, social learning, Bayesian modeling, sociophonetics, word learning, epistemic trust

## Abstract

We incorporate social reasoning about groups of informants into a model of word learning, and show that the model accounts for infant looking behavior in tasks of both word learning and recognition. Simulation 1 models an experiment where 16-month-old infants saw familiar objects labeled either correctly or incorrectly, by either adults or audio talkers. Simulation 2 reinterprets puzzling data from the Switch task, an audiovisual habituation procedure wherein infants are tested on familiarized associations between novel objects and labels. Eight-month-olds outperform 14-month-olds on the Switch task when required to distinguish labels that are minimal pairs (e.g., “buk” and “puk”), but 14-month-olds' performance is improved by habituation stimuli featuring multiple talkers. Our modeling results support the hypothesis that beliefs about knowledgeability and group membership guide infant looking behavior in both tasks. These results show that social and linguistic development interact in non-trivial ways, and that social categorization findings in developmental psychology could have substantial implications for understanding linguistic development in realistic settings where talkers vary according to observable features correlated with social groupings, including linguistic, ethnic, and gendered groups.

## 1. Introduction

Common wisdom among adults when listening to speech is to “consider the source.” The identity of a speaker can provide a wealth of context in interpreting a speech act. Despite this, social reasoning about differences between sources has not heretofore been considered part of early word learning, and is not traditionally included in computational models of infant word learning (e.g., Pinker, 1979; Chater and Manning, 2006; Frank et al., 2009). These models assume that the linguistic content of speech may be characterized without respect to the identity of the speaker. Effectively, this equates to an assumption that learners trust data from all informants equally, and that all informants are equally likely to be speakers of the same dialect. Learners in these models are defined without sociolinguistic or metalinguistic awareness, and without the ability to socially differentiate categories of informants. Instead, the models effectively describe infants as acquiring a single target dialect which is the only linguistic code available in the environment.

The omission of sociolinguistic variation from previous computational models makes them unable to capture effects of non-linguistic social and cultural associations on language learners' responses to linguistic data. It also prevents them from capturing effects of learners' perception of linguistic group membership on the perception of labels. These factors are known to impact adult linguistic perception: adults perceive typological distinctions between language varieties, such as regional accent or dialect, and have concomitant beliefs about the social significance of these variations being deployed. There is also substantial evidence that adult knowledge of accent and dialect groups impacts speech perception and perceptual learning of speech contrasts (Clopper and Pisoni, 2004). To understand language acquisition we must begin to confront the true complexity of the learning problem, but these simplifying assumptions ultimately render previous models insufficient for investigating effects of social variation on language acquisition.

This paper introduces a model which incrementally departs from key simplifying assumptions in previous models by incorporating existing understandings of infant social evaluation from literature in developmental psychology. Our model instantiates a learner who is acquiring their target dialect in the presence of a single available alternative which has greater variation. Therefore, our listener must be selective in their attention to linguistic informants. To learn their target dialect, the learner must also *avoid* natively acquiring any non-target dialects, a task which requires selective trust in informants as sources of linguistic knowledge, and which learners may simplify by recruiting social beliefs about the relationship between dialect group membership, social group membership, and source quality. We demonstrate that the addition of inferences about talker social and dialect group membership to the speech perception task defines the language learning problem in a way that more effectively and parsimoniously explains and contextualizes existing findings in the literature on infant speech perception.

Our model accounts for infant behavior in response to social agents of varying perceived reliability. This aspect of informant perception is only one element in a full account of early word learning. The work presented here is compatible with existing theories of language learning which have incorporated social cognitive skills in describing the language acquisition process. However, these theoretical models have not directly implemented mechanisms to account for categorical perception of social or dialect groups. Models which integrate acoustic perception and social cognitive skills can be further extended by positing that children may apply their skills differently in response to perceived differences between informants. By treating person perception as a component of speech perception, our model is capable of predicting differences in a listener's responses to informants who are engaged in behaviors judged by adults to be linguistically equivalent, but who have distinctive physical appearances or speech accents. Unlike previous acquisition models, the epistemic trust model presented here accounts for infant behavior in response to social agents of varying perceived reliability, allowing us to pose questions about the role learners' non-linguistic perception of identity plays in the speech perception task.

Moreover, in defining more than one variety of speech, and associations between these varieties and non-linguistic affiliations, we can describe the learner as possessing a metalinguistic awareness in the form of confidence regarding the accuracy of linguistic judgements made under different social circumstances. Supposing that the learner must perceive some nominal social significance in the variation they acquire also allows our model to describe listeners as making distinctions between talkers on the basis of their speech behavior. This framework extends the definition of the language acquisition task to necessarily incorporate processes of person perception, which are predicted to have cascading effects on listeners' attention to both linguistic and non-linguistic behaviors. Further, the model can predict that learners will exhibit metalinguistic judgements regarding the relative quality of an informant's speech behaviors, reserving the most scrutiny for informants belonging to groups which are believed knowledgeable.

## 2. Background

There is a wealth of evidence showing that beliefs about social variation affect adult perception of speech. Adults associate linguistic variation not only with geographical regions but also with numerous socially constructed categories, including genders, sexual orientations, socioeconomic classes and personas (D'onofrio, 2018). Social beliefs influence how adults recognize and interpret speech. For example, Johnson et al. (1999) presented adult listeners with tokens from a synthesized phonetic continuum, and found that the presentation of female faces caused participants to perceive a shifted category boundary, with the effect being strongest for faces which were rated as more stereotypically feminine. These results demonstrate that adults may interpret the same exact speech signal differently depending on the cues to social groupings the listeners receive in advance. Similar effects can be obtained without providing an image of the talker. The listener's linguistic interpretations may also be manipulated with cues and instructions that are merely suggestive of talker features, including age (Hay et al., 2006b), sex (Johnson et al., 1999), and geographical dialect group (Hay et al., 2006a).

Despite our rich knowledge of adult perception, there has been little research on what cognitive building blocks are used to make sense of socially conditioned linguistic variation, and particularly how these building blocks may develop in infancy. Theoretical models such as PRIMIR and the emergentist coalition-model, which stress the importance of social cognitive processes in language acquisition, importantly do not define contrastive roles for social agents based on judgements of their epistemic reliability (Hirsh-Pasek et al., 2000; Werker and Curtin, 2005). The model presented here is compatible with these accounts, and can extend these theories by predicting how infants may respond contrastively to distinctive informants engaged in identical behaviors.

Models of language acquisition which entirely abstract away from social variation in speech have no way to explain how or when socially conditioned perceptual differences emerge. Here we draw on the literature on epistemic trust as a simple and useful starting point for more effectively integrating our knowledge of infant social and linguistic development.

Epistemic trust is the process by which a learner uses direct observations to infer which sources of information are trustworthy. Evidence from non-linguistic domains indicates that infants perceive both non-linguistic behavior and group membership as relevant to judgements of informant quality in non-linguistic tasks. Infants are more likely to imitate instrumental actions when they are presented with informants who demonstrate competence in the conventional usage of objects (Zmyj et al., 2010), reliability with respect to eye gaze (Tummeltshammer et al., 2014), and emotional cues (Poulin-Dubois et al., 2011). Xiao et al. (2018) compared the responses of 7-month-old infants to talkers of two different racial groups whose eye gaze either more or less reliably predicted the appearance of a stimulus at the indicated location. When the talker's gaze was perfectly reliable, cuing the stimulus 100% of the time, infants were significantly above chance in following the talker's gaze regardless of that talker's racial group. However, when the gaze cue was unreliable, only cuing the stimulus 50% of the time, infants were significantly more likely to follow the gaze of the own-race talker compared to the other-race talker. Given uncertainty about the significance of the informant's behavior, infants appeared to recruit prior beliefs about the relationship between non-linguistic social cues and informant trustworthiness. These kinds of social evaluations may constitute an overlooked source of insight into infant behavior on word learning tasks.

There is also significant evidence that infants learn more effectively from familiar speakers compared to novel speakers (Parise and Csibra, 2012; Fennell and Byers-Heinlein, 2014; van Rooijen et al., 2019). This advantage may be explained with respect to acoustic experience, but may also be interpreted as an effect of epistemic trust. Under the latter account, infants' preferential attention to familiar speakers is borne of their social perception that these speakers are comparatively reliable. Beliefs about epistemic trust grounded in perception of social groups provide a way to extend existing word learning models, situating the predictions they make about word learning behavior relative to explicitly defined and testable hypotheses about not just linguistic, but social objectives.

There is one previous model that has linked epistemic trust to word learning behavior in older children. Shafto et al. (2012) uses epistemic trust to simulate the behavior of the preschool age children on a word learning task. Supposing that the utility of linguistic data is not uniformly consistent across informants supplying those data, they then describe this utility as a probabilistically defined function of the informants' qualities. In other words, some informants will have speech with superior accuracy, and some informants will have qualities associated with superior accuracy. Learners may infer the presence or absence of latent shared intentions between informants, and use these inferences to selectively guide their attention.

In a study by Corriveau et al. (2009), 4- and 5-year-old children were presented with a display of unfamiliar adults labeling unfamiliar objects. The children were then asked to choose an adult to endorse a label for yet another novel object. When the children observed all but one of the adults agreeing on object labels, they preferred to endorse labels from adults who they had observed participating in the consensus, as opposed to those who they had seen dissenting. Since both the labeled objects and the speakers supplying the labels were unfamiliar to the experimental participants, they could not have been relying on prior knowledge of either when selecting labels to endorse; instead, observing an informant participating in a consensus was enough to influence the children's perception of their testimony.

Children preferred to answer the question “Which is the [novel object label]?” with information given by an informant who had previously agreed with a majority, over information from an informant who had dissented. Shafto et al. (2012) successfully modeled this result by asserting that children preferentially attend to the most epistemically reliable *individual* linguistic sources when learning words. Their model was designed to model the behavior of 4- and 5-year-old children, but based on the literature on infants' judgments of epistemic trust reviewed above, there is reason to believe that infants rely on similar socially motivated inferences to guide their processing of speech at a much earlier age.

Moreover, drawing on the literature on non-linguistic social learning, we suggest that infant language learners may fruitfully bring their social knowledge to bear on linguistic tasks. Contrary to the assumptions of previous computational models of early language learning that have largely omitted social perception, it is well-accepted that linguistic and non-linguistic judgments of informants are tightly linked.

For example, studies of the ontogeny of attitudes toward in-group and out-group members (e.g., Mahajan and Wynn, 2012; Buttelmann and Böhm, 2014) are predicated on the assumption that socially motivated cognitive grouping is part of human behavior and likely emerges quite early. Judgments of an informant's value as both a linguistic and a non-linguistic source of information are tightly linked in infants (Kinzler et al., 2007; Schachner and Hannon, 2011). Infants are more likely to imitate non-linguistic behavior and learn novel words from informants who they have observed accurately labeling familiar objects, compared to informants who they have observed using familiar labels inaccurately (Poulin-Dubois et al., 2011; Brooker and Poulin-Dubois, 2013). Liberman et al. (2017) familiarized 9-month old infants with videos of two people who spoke the same or different languages, and found that the infants looked longest to subsequent videos which showed the people who did not share a language affiliating.

Given that infants use both epistemic trust and linguistic behavior to reason about social groups, it is possible that social knowledge may have a reciprocal effect on measures of their linguistic perception. Preverbal infants, including newborns, use non-linguistic observations to discriminate between social partners (Akhtar and Gernsbacher, 2008; Coulon et al., 2011; Maurer and Werker, 2014; Cirelli et al., 2016). Diesendruck et al. (2010) provide evidence that older children distinguish between informants who have demonstrated knowledgeability of conventional label forms and those who have not, more often expecting talkers who have used typical labels to also obey familiar pragmatic conventions for referencing objects. The children expect talkers who show knowledge of the conventions to consistently obey them, but do not extend these expectations to informants who have demonstrated unconventionality in their labeling behavior. This study demonstrates that learners may use metalinguistic judgements of conformity to guide their linguistic expectations of talkers. We hypothesize that infants may be similarly influenced by metalinguistic perceptions of comparative speech quality in the context of a salient social group.

Supposing that social groups may also have *characteristic linguistic behaviors*, infants' ability to distinguish between informants based on non-linguistic features and behaviors may assist them in identifying and attending to socially meaningful linguistic distinctions. However, there are no existing models of speech perception that incorporate both epistemic trust and listener beliefs about social groups.

The model in Shafto et al. (2012) describes a learner who is sampling from linguistic sources who may be described individually as exhibiting more or less helpful intentions. However, this model still falls short of making a principled connection between infant expectations of linguistic and non-linguistic behavior. Instead of describing a learner who is sampling from linguistic sources whose speech individually may or may not exhibit helpful intentions, we wish to define a learner who also has expectations about the helpfulness of an informant based on non-linguistic observations. The learner's task, then, is still to infer the correct linguistic categories under uncertainty about the quality of the speakers, but now with the additional aid of beliefs which allow them to a priori categorize informants as more or less likely to have helpful intentions based on their putative affiliation with a social group.

The model introduced in the next section describes infants as not only attending to more knowledgeable talkers, but also as attending more to talkers from *characteristically knowledgeable groups*. This new model defines a listener who is capable of reasoning about the talker's membership in both linguistically and non-linguistically defined groups, allowing us to explore how joint perception of cues which indicate shared identities between talkers affects listeners' performance in word learning tasks. Crucially, the model presented here positions speech itself as an affiliative cue, allowing us to characterize the learner's task as preferentially learning the linguistic forms of their own social group. This choice to characterize speech as implicitly associated with social groups represents a significant departure from previous models of language learning.

To model listeners as evaluating variation between speakers for social value, we characterize talkers as exhibiting one of two labeling behaviors: knowledgeable labeling, which consistently has correct form and substance, and unknowledgeable labeling behavior, which does not. Imputing relative knowledgeability allows us to model variation between speakers as more or less desirable, or socially meaningful. Using this new model, we show that there is already evidence that social inference influences early word learning. We show this model can parsimoniously predict effects of variation in the social status of label sources on infants' looking to both labeled objects and people. Further, we show that this effect can explain infant behavior both when the informant's knowledgeability and group membership are apparent, and when these variables are the subject of inference under uncertainty.

The binary contrast we investigate between knowledgeability and unknowledgeability represents a stark simplification of what is known about how infants track informant reliability. However, the present paper offers an initial step in computationally approaching the effect of social knowledge on label learning by positing that learners have attentional preferences for reliable *kinds* of social agents. In effect, previous models have defined a single kind of linguistic listening and learning, predicting that infants will apply this approach uniformly to linguistic data. The model presented here predicts that non-linguistic perception of informants as belonging to categorical kinds will produce metalinguistic perception of an informant's speech quality with respect to their group membership. We demonstrate that metalinguistic expectations of correlations between speech behavior and talker identity can predict infant looking behavior in two experiments.

## 3. Model

We hypothesize that developmental changes in infant social perception may account for findings which have previously been interpreted to indicate asocial linguistic learning. To test this hypothesis, we compare a model developed to describe social word learning with an asocial word learning model, and contrast their ability to account for infant looking behavior in two experimental tasks.

Our model builds on the model proposed by Shafto et al. (2012), but is distinct in two ways. Firstly, we assume that the knowledgeability of an informant is a function of their group membership. Secondly, we simplify the learner's problem by excluding the possibility of knowledgeable, intentionally deceptive informants. Learners must instead compare informants who are assumed to be at least minimally helpful, with none actively hindering the learner.

To model the learning problem, we assume that all informants speak the target language, and the learner's task is determining which informants are more helpful for the purpose of learning object labels in the target dialect. In the following sections we demonstrate that this model can predict the pattern of results in two experiments investigating infant word learning: a familiar word recognition task (Koenig and Echols, 2003), and a novel word learning task (Rost and McMurray, 2009).

**Figure 1:**
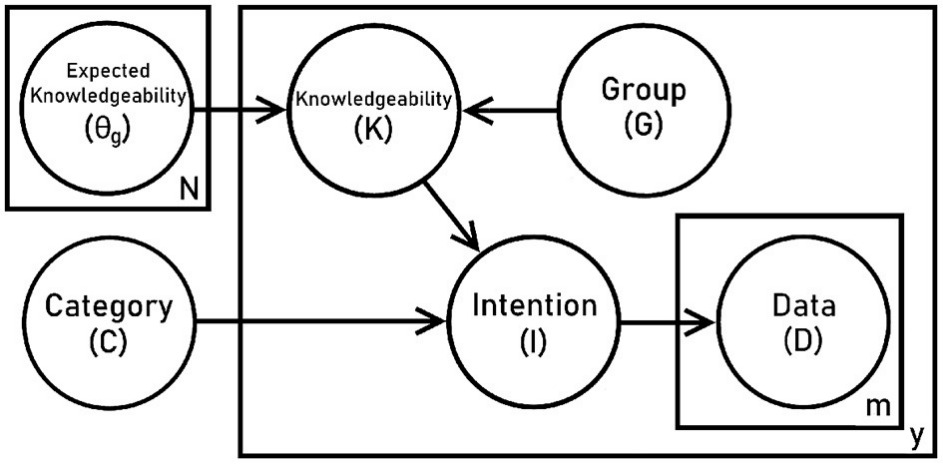
Graphical model for simulating word listening tasks.

Figure 1 shows our graphical model. We suppose that each speaker may be described with two characteristics: their group membership *G*, and their individual knowledgeability *K*. The parameter θ_*g*_ defines the characteristic distribution of knowledgeable informants for a given group,

(1) $$\begin{array}{r} p\left(K|G\right)=\text{Bernoulli}\left(\theta_{g}\right) \end{array}$$

The speaker characteristic of knowledgeability encodes how likely it is that the speaker knows the correct label for the object, defined by the linguistic category *C*. Given the correct linguistic category, and the speaker's knowledgeability, the speaker then forms an intention *I* to produce speech data *D* which may or may not be a correct pronunciation of the label. For example, supposing that the object being labeled is a dog, the infant will recognize multiple variant pronunciations as referring to this object, (e.g., both /dɔg/ and /dəg/) but may only identify one pronunciation as the correct form for their dialect.

We present a simple case, assuming that there are only two levels of knowledgeability: knowledgeable speakers always intend to produce the correct label, whereas only a fraction of unknowledgeable speakers do so. However, both kinds of informants are consistent in the labels they produce. They intend to produce the same label each time they label the same object. In other words,

(2) $$\begin{array}{r} p\left(I|C,K=1\right)=\delta_{IC}\\ p\left(I|C,K=0\right)=\frac{1}{n} \end{array}$$

where δ is the Kronecker delta function, taking value one when *I* matches *C* and zero otherwise, and *n* is the number of possible labels. For the simulations in this paper, we assume that the data *D* that the speaker produces always perfectly matches the speaker's intention *I*, meaning that data are also distributed as

(3) $$\begin{array}{r} p\left(D|C,K=1\right)=\delta_{DC}\\ p\left(D|C,K=0\right)=\frac{1}{n} \end{array}$$

While there is no practical distinction between *I* and *D* in our simulations, we retain this distinction in our model in anticipation that it will be useful for modeling cases of ambiguous and errorful pronunciation in future work.

Although the model employs a binary contrast between knowledgeable and unknowledgeable speakers, it is possible that children make finer-grained distinctions between degrees of knowledgeability. Nonetheless, this simple binary contrast between knowledgeable and unknowledgeable speakers should be considered compelling, as it illustrates that even with the minimum number of speaker states under consideration, task performance can be predicted by predicting how the listener socially differentiates speakers. We show that even this elementary binary distinction between knowledgeable and unknowledgeable speakers allows us to capture effects that previous models are unable to capture. Future work could investigate more complex perceptions by representing knowledgeability as a continuous variable ranging from fully knowledgeable to fully unknowledgeable, or by representing perceived knowledgeability as a more nuanced set of variables indexing beliefs about knowledgeability which differ between contexts.

In effect, the model posits that knowledgeable speakers categorically give trustworthy testimony, and variation in form strictly occurs between unknowledgeable informants. Knowledgeable speakers are defined as homogeneously predictable and undifferentiated with respect to labeling behavior. By contrast, unknowledgeable speakers display characteristic patterns of errors which allow most to be differentiated from knowledgeable speakers by their labeling behavior. This means that the model describes infants as receiving a mixture of three kinds of testimony: correct testimony from knowledgeable sources, correct testimony from unknowledgeable sources, and incorrect testimony from unknowledgeable sources.

This model instantiates epistemic trust by defining the learner as preferring speech sources who are more knowledgeable, and therefore more likely to produce correct labels. Learning labels then relies on a preference for speech events which are informative with respect to the informant's knowledgeability. We then presume that these kinds of speech events may be distributed differently in different groups of talkers. The learner's level of epistemic trust for any given individual is therefore predictable based on that individual's group membership. By characterizing the listener's beliefs about kinds of talkers and the kinds of speech variation characteristically associated with them, we can model the listener as using this knowledge of variation to guide their attention to both labels and the sources which produce them.

If infants rely on beliefs about group membership and characteristic knowledgeability to guide their interpretation of labeling events, we should expect experimental conditions wherein informants are clearly knowledgeable to elicit greater looking times than conditions where informants are apparently unknowledgeable. Supposing that infants can readily distinguish the two types of labeling sources, and reason about which informants' speech is characteristically more useful for the purpose of language learning, perception of non-linguistic differences between informants may deeply impact word learning.

The inclusion of knowledgeability and of groups of informants in this model changes the nature of the word learning problem, relative to what had been assumed in previous models. It contextualizes word learning to a particular group of informants speaking a particular dialect, so that there is no longer just one language in the input, and just one correct label for an object. Instead, there is variation in labels, and only some of the variation corresponds to the learner's target dialect. Although human infants can acquire multiple dialects, and can perceive distinctions between social and dialect grouping, the learner modeled here supposes that social groups have predictable relationships to dialect groups. This model provides a step toward modeling multilingual learners by positing that infants must be able to effectively navigate an input containing two dialects, and use speaker identity to determine when attention to each dialect is appropriate. This enables behavior on tests of lexical knowledge to be described as influenced by beliefs about dialect and social groups. Attentional biases regarding perceived *kinds* of talkers may allow infants to use non-linguistic social judgements as a proxy for evaluating the informational content of speech.

In two simulations, we show that this inclusion of social information in a model of word learning is crucial for capturing infant behavior on tests of word recognition and word learning. While previous work had already hypothesized that infants attend to social information, we show how this information may be closely intertwined with core inferences in word recognition and learning, suggesting that it is impossible to understand the inferences infants' make when learning their first words without also understanding the role of social information in that learning process. Our computational framework introduces this social context into a model of early word learning for the first time; we show that it captures existing results, and discuss ways that it can facilitate future research on word learning in social contexts.

## 4. Simulation 1

### 4.1. Task: Listening to Familiar Words

Simulation 1 uses our model to simulate a word recognition task. Koenig and Echols (2003) compared the responses of 16-month old infants to true and false labeling events provided by either live human experimenters or inanimate audio speakers. They found that in response to incorrect labeling events, these infants showed longer looking times to the source of the label when it was a human experimenter. However, they did not look longer to an audio speaker when it was the source of the incorrect object labels. In this scenario, the infants apparently attend to the type of speech source: whether it is a human experimenter or an inanimate speaker, and accordingly have differing expectations about whether the informant will be a reliable source of object labels.

The 16-month old infants were presented with photographic color slides of five familiar objects: a chair, duck, cat, ball, and shoe. As each image was displayed, the informant provided a label for it by reporting “That's a _.” In the control condition, all of the labeling events were correct, matching the displayed objects. In the test condition, all of the labels were false. For example, while a picture of a cat is displayed the infant might hear “That's a shoe.” Researchers coded the infants' behavior, measuring the amount of time they spent looking to both the displayed object, the sources of the labels, and to their caregiver. In Experiment 1, the infants heard the labels from a human experimenter seated next to them. In Experiment 2, the labels were provided by an audio speaker placed in the same location.

The researchers hypothesized that the infants' attention to the source would be influenced both by the accuracy of the label and the type of source providing it. Indeed, they found a broad effect of label accuracy: the infants looked longer at the object when hearing true labels, than when hearing false labels. They also found an effect of label source: the infants looked longer to both objects and label sources when the label sources were human speakers than when they were audio speakers. Lastly, there was an interaction of these two effects: infants looked longer to their caregivers and to the human speakers when labels were false rather than when they were true, but within the audio speaker condition, their looking to the label source was not significantly affected by accuracy.

**Figure 2:**
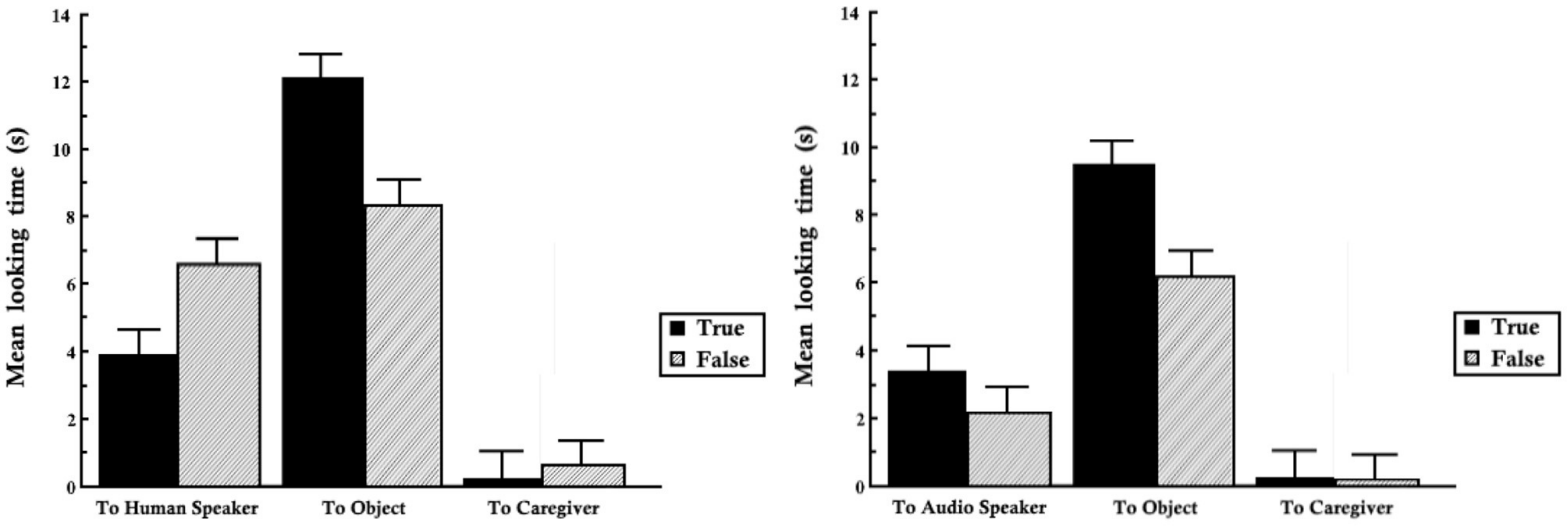
Looking time to parent, label source, and label target for true and false labels from human (experiment one, **left**) and audio speaker (experiment one, **right**).

Overall, infants looked more to both the object and the speaker in the human labeler condition, as shown by the total area of the bars in the graph on the left of Figure 2. Only average looking time was reported, so we are unable to analyze the time course of this data. Comparatively, the total looking time to both the labeler and the object is lower in the audio speaker labeler condition. Provided some certainty that the labeling source belongs to a group which is likely to be unknowledgeable (audio speakers), we will show that a Bayesian model predicts the infant should find correct labeling events from this source more surprising than incorrect labeling events from this source. Likewise, within the condition where the source belongs to a group which is accurately assessed as likely to be knowledgeable (adults) then we expect the infant to find incorrect labeling events more surprising.

In other words, the pattern of looking times corresponds with the infants having a high degree of confidence in their prior beliefs that speech from adults is likely to be much more reliably informative than speech from audio speakers. Koenig and Echols (2003) also included a third experiment to ensure that the difference in infant responses was a result of infants interpreting the human as a labeling source, and not just an effect of their presence. In the presence of a silent human experimenter, infants tended to look away from the object longer during false labeling event without choosing to attend to the silent human. These results demonstrate that the infants' looking behavior is influenced by perceptions which are specific to the speaker.

### 4.2. Model

We use our model to simulate infants' behavior in the experiments from Koenig and Echols (2003) and show that infants' behavior can be modeled as the result of reasoning about which informants' speech is characteristically more or less useful for the purpose of language learning. We frame infants' speaker perception as an inference on the knowledgeability, *K*, of the speaker, and contrast expectations about the effect of speaker perception on infant looking behavior in two conditions: with and without the production of labels.

To simulate these data we model the category *C* as known, corresponding with the infant's access to the visual object display and lack of inconsistent perceptual access cues. The learner is modeled as inferring the speaker's knowledgeability *K*. Infants are assumed to perceive the group *G* accurately—adult human or audio speaker—and to already know the θ_*g*_ associated with that group.

In the context of labeling, we expect human sources to be experts while audio speakers are less reliable. In our simulation, this assumption can be operationalized by setting the parameters such that adult informants are very likely knowledgeable, while audio speakers are less likely to be knowledgeable, e.g., *P*(*K* = 1|*G* = *adult*) = θ_*adult*_ > *P*(*K* = 1|*G* = *audio*) = θ_*audio*_. From the parameters θ_*g*_ we may predict that on average, a more knowledgeable type of speaker will provide more information about an object whose label is unknown than a less knowledgeable type of speaker. However, given that infants already knew the labels for all of these objects, we focus on the value of each type of informant for a different inference problem: inferring the speaker quality *K*.

When the learner observes a label, the impact on their certainty about the labeler's knowledgeability will be different depending on their beliefs about the affiliation of the speaker and the characteristic knowledgeability of their social group. We model this with a measure of information, the Kullback-Leibler divergence, which describes how differently knowledgeability is expected to be distributed before and after observing a label *D*, in the context of a correct label *C* and talker affiliation with group *G*. Equation (4) below describes this measure, and further details are given in Appendix 1.

(4) $$\begin{array}{l} D_{KL}\left(P\left(K|D,G,C\right)||P\left(K|G,C\right)\right)\\ \;=\underset{K}{\sum }P\left(K|D,G,C\right)\log\frac{P\left(K|D,G,C\right)}{P\left(K|G,C\right)} \end{array}$$

We predict that the total looking time will reflect infants' interest in a stimulus, which we index here as relative entropy (KL-divergence). While newer eye-tracking methods do allow tracking of continuous looking behavior and individual fixations, these details were not included in the data we are modeling.

We posit that knowledgeable speakers never produce an incorrect label. This means that on average, data from members of typically knowledgeable social groups—like humans—carry more information about the individual speaker's knowledgeability than data from members of typically unknowledgeable groups do. Moreover, infant looking time to talkers who have provided labels is predicted to be highest for adults labeling objects incorrectly, because this is the most informative scenario regarding informant knowledgeability: an informant that the infant had predicted to be almost certainly knowledgeable, is now revealed to be unknowledgeable. Such data defy the infant's prior expectations both that the informant is knowledgeable, and that their testimony will therefore be correct. This simple model does not include belief updating, but instead predicts that the infant will treat informants as equally unfamiliar on each trial.

### 4.3. Results

We applied our computational model to simulate infant looking behavior to social actors in response to true and false labels. The black bars in Figure 2 show the amount of time infants spent looking in response to true labels, while the gray bars measure looks in responses to false labels. Both audio speakers and humans elicited more looks to objects with true labels, and accordingly the black bars in the center of each graph are longer than the gray bars paired with them. Given that infants already know the correct labels of these objects, and given the well-established finding that infants look longer toward the object being labeled in preferential looking paradigms, this benefit of true labels over false labels would be predicted under many different accounts, including those that do not incorporate source evaluation. However, infant looks to the label sources show a different pattern which cannot be explained by a preference for correct label forms.

Infants looked significantly longer to label sources in the condition where they were human speakers producing false labels than inhuman speakers providing correct labels, as shown by the leftmost gray bar in Figure 2. The third pair of bars in this left figure shows the same pattern. When infants heard false labels from adult speakers they also looked significantly longer to their caregiver, whose lap they were seated on. However, contrasting true and false labels from audio speakers produces no significant difference in the infant looks to the labeler and caregiver.

Our model can reproduce this qualitative pattern of looking behavior toward the label sources. Table 1 describes the relative entropy (KL divergence between prior and posterior probability) of the speaker's knowledgeability given four possible circumstances of the labeling utterance—that the speech is either unambiguously supportive (a true label) or contradictory (a false label) to the given category, and that the speaker is either an adult or an audio speaker, for an illustrative set of model parameters: θ_*audio*_ = 0.15 and θ_*adult*_ = 0.85.

**Table 1:** KL divergence of K with D compared to without for each type of observation.

**Condition**	**D_*KL*_(*P*(*K*|*D, G, C*)||*P*(*K*|*G, C*))**
Correct adult	0.0846
Incorrect adult	2.3219
Correct audio speaker	0.1701
Incorrect audio speaker	0.2345

The incorrect adult informant described in the second row of Table 1 is expected to provide far more information about knowledgeability than any other type of speaker. Even without a bias to perceive adults as more likely to be speech agents than audio speakers, the belief that an adult using incorrect labels is a surprisingly unrepresentative member of a characteristically knowledgeable group can explain infants' predisposition to attend to these informants far longer than others. This pattern is consistent with the experimental results that infants looked longest to these types of labelers.

**Figure 3:**
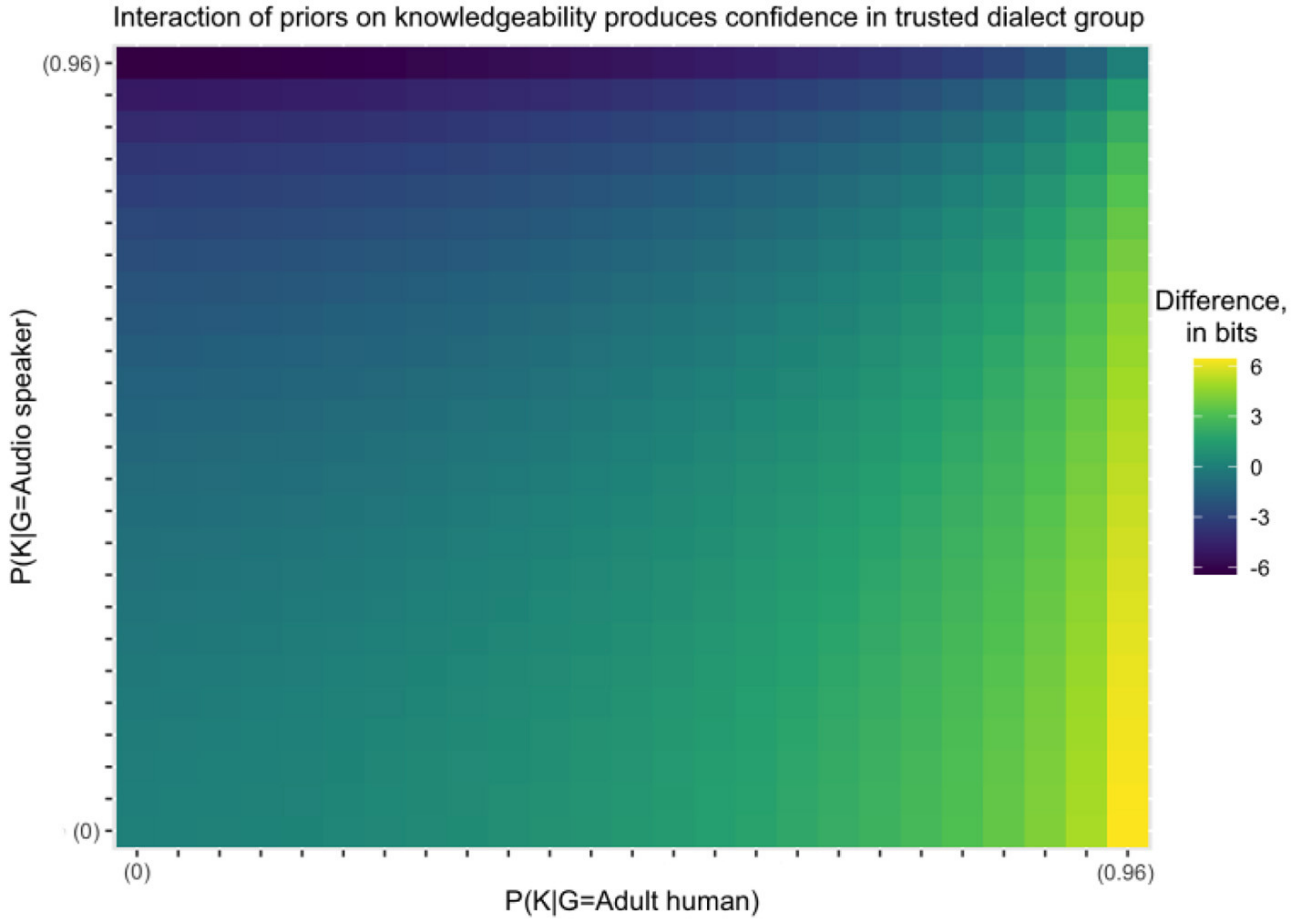
The difference between the KL divergence for adult humans given incorrect and correct data, less the difference between the KL divergence for audio speakers given incorrect and correct data. The axes on the graph span most of the range between 0 and 1, but omit the last interval because KL divergence after hearing incorrect data is undefined when the prior on knowledgeability is 1.

Figure 3 shows how these predictions change for different values of the model parameters. The interaction effect, in which infants look longest to incorrect adult informants, is summarized by taking the model's predicted difference in looking between incorrect and correct humans, and subtracting the predicted difference in looking between correct and incorrect audio speakers. Lighter colors indicate more looking to adult humans, and darker colors indicate more looking to audio speakers. Essentially, the color captures the size and direction of the interaction effect.

For all scenarios where the priors on the knowledgeability for humans and audio speakers are identical, the difference in the KL divergence for correct vs. incorrect labels is also identical across the two types of informants. This equality can be observed in the identical hue in each box along the main diagonal from bottom left to top right. This means that a model that does not distinguish between types of informants—and thus assumes an equivalent prior on knowledgeability for both—would predict that infants exhibit the same looking behavior toward humans and audio speakers.

The empirical data instead show an interaction in which infants looked much longer to the incorrect human than to the correct human, but showed approximately equal looking to the incorrect and correct audiospeakers. This direction of interaction is shown as lighter colors on the graph, which occur below the main diagonal, and particularly for high priors on human knowledgeability. The model predicts that infants will look longest to incorrect adult humans so long as they believe that human adults are, as a class, the kind of informant which is *most likely* to correctly produce the target dialect. Individuals with apparently deviant behavior are predicted to provide the most informative testimony with respect to the correct priors on knowledgeability, as we have defined unknowledgeable speakers to be unpredictable compared to knowledgeable ones.

There is small difference in Table 1 between correct and incorrect labels from audio speakers in our simulation that goes in the opposite direction from the trend found in the data. This difference may not be meaningful, however, given that it was not statistically reliable in the empirical data. The size of this predicted effect in the model depends on the prior on audio speaker knowledgability; as the prior gets smaller, the effect is also predicted to get closer to zero (reaching zero when θ_*audio*_ = 0). No parameter setting in the model predicts an effect in the other direction. Nevertheless, small predicted effects in the model are still compatible with non-significant experimental results, given that experiments do not always have enough statistical power to reveal effects of small magnitude. The key result is the much larger predicted difference in looking times between correct and incorrect labels when the informant is expected to be knowledgeable.

### 4.4. Discussion

In our simulation, the relative entropy of infants' beliefs before and after hearing a label closely mirrored their looking behavior toward label sources. These results confirm the importance of perception of group membership on infants' behavior. The differences in looking behavior in response to humans and audio speakers fall straightforwardly out of a model where these different groups of speakers have different characteristic levels of knowledgeability. While the original model from Shafto et al. (2012) captures inferences about the knowledgeability of individual informants, the inclusion of groups in our model was crucial for reproducing infants' behavior: it provides a prior distribution over knowledgeability that differs between the two experimental conditions. A model that did not distinguish between groups of informants would incorrectly predict identical patterns of infant looking behavior for both human and inanimate speech sources. These findings suggest that beliefs about social groups play a role in lexical learning even in very young infants' word recognition processes.

Infants' looking behavior toward the social actors when they provide incorrect labels is crucial to distinguishing our account from alternatives. Although across the conditions, infants' overall looking to both social actors and objects is higher when adults supply the labels, this pattern could be evidence either by a preference for the adult labelers or a dispreference for the audio speakers, without necessarily requiring inferences about knowledgeability. However, the prior belief that adult labelers are more trustworthy, and can be expected to more often provide correct labels, predicts both the overall pattern of longer looking in response to adult labels, and the significantly longer looking times to both the adult labeler and caregiver in response to incorrect labels.

While the two “social groups” in this experiment were humans and audio speakers, children are likely to have a more general ability to sort informants into social groups that have different characteristic levels of knowledgeability about the target dialect. This could have far-reaching implications. For example, Supposing that evaluation of informant knowledgeability incorporates both linguistic and non-linguistic social perception, the function of attentional and memory processes controlling lexical-phonetic perception in learners is predicted to vary even among speakers who share a language, by virtue of the distinct social beliefs entailed by membership in different social groups defined by dialect. If children expect membership in socially defined groups to predict the form and meaning of speech, we should predict differences in performance on tests of language knowledge by children with distinct social backgrounds.

Perception of non-linguistically defined groups and informant states, such as perceptual access cues, may also influence learners' attention to linguistic cues and their beliefs about them. Koenig and Echols (2003) included an experiment with both a silent human and an audio speaker to confirm that the longer looking times in response to adult labels were not simply driven by the physical presence of the human labeler, and found that there was a marginally significant effect on looking to the object, but no significant effect on looking to the labeler or the caregiver. In a fourth experiment, infants were presented with a human labeler who faced away from the visual display. Again, no significant effect was found for looks to the caregiver, however they did find an effect of condition—infants tended to look longer to the backwards facing human labeler for true labels. These results are consistent with our hypothesis that while infants assume that human informants as a group are more knowledgeable than audio speakers, their responses to labels are also influenced by sophisticated reasoning about the knowledgeability of individual informants.

The binary contrast used in this model is a simplification. Many experiments have shown infants are capable of acquiring labels from audio speakers. However, there is also significant evidence that infants' social abilities are intrinsic to natural language learning. For example, infants who receive live exposure to a phonetic contrast, when tested on the contrast, outperform infants exposed via digital displays Kuhl (2007). Although we predict that infants will more readily learn from live human sources compared to audio speakers, we do not predict that infants cannot learn from dispreferred sources, but rather that infants *expect to learn more* from the testimony of preferred sources and will be comparatively hyperattentive to deviance among informants of preferred social categories.

Our model is compatible with theoretical accounts of cross-situational word learning which seek to integrate infant attention to acoustic and social cues. However, our model further posits a mechanism to specifically explain how children acquire the ability to interpret linguistic variation in the context of social *identities* in addition to social behaviors. Demonstrating this experimentally requires studies which contrast informants in social identity and epistemic reliability.

In the next section we provide further evidence in support of our model, applying it to describe infants' behavior on a word learning task, where the label for an object is not known in advance. Novel labels preclude infants from using their lexical knowledge to make judgements about the knowledgeability of individual speakers based solely on those speakers' own utterances. We show that we can nevertheless model infants' behavior by assuming that they use agreement between talkers to make inferences about individual speaker's knowledgeability. Thus, social inference appears to play a key role in infants' lexical processing, even when infants are still learning the meanings of the words that they hear.

## 5. Simulation 2

### 5.1. Task: Listening to Novel Words

Simulation 2 uses our model to simulate a novel word learning task. A large body of work has used an audiovisual habituation experiment called the Switch Task to investigate early word learning. Results from Switch task experiments show that infants who can perform well on a task discriminating two lexical neighbors, or words which differ by a single phoneme (e.g., “buk” and “puk”), nevertheless do not consistently discriminate those same labels after being habituated to the presentation of these speech tokens as the labels of two different objects (Stager and Werker, 1997). This difficulty does not appear for pairs of words which differ by multiple phonemes (e.g., “lif” and “neem”) (Werker et al., 1998). Slightly older infants show significantly improved performance, with 17-month-old infants being successful at learning the phonetically similar words (Werker et al., 2002).

**Figure 4:**
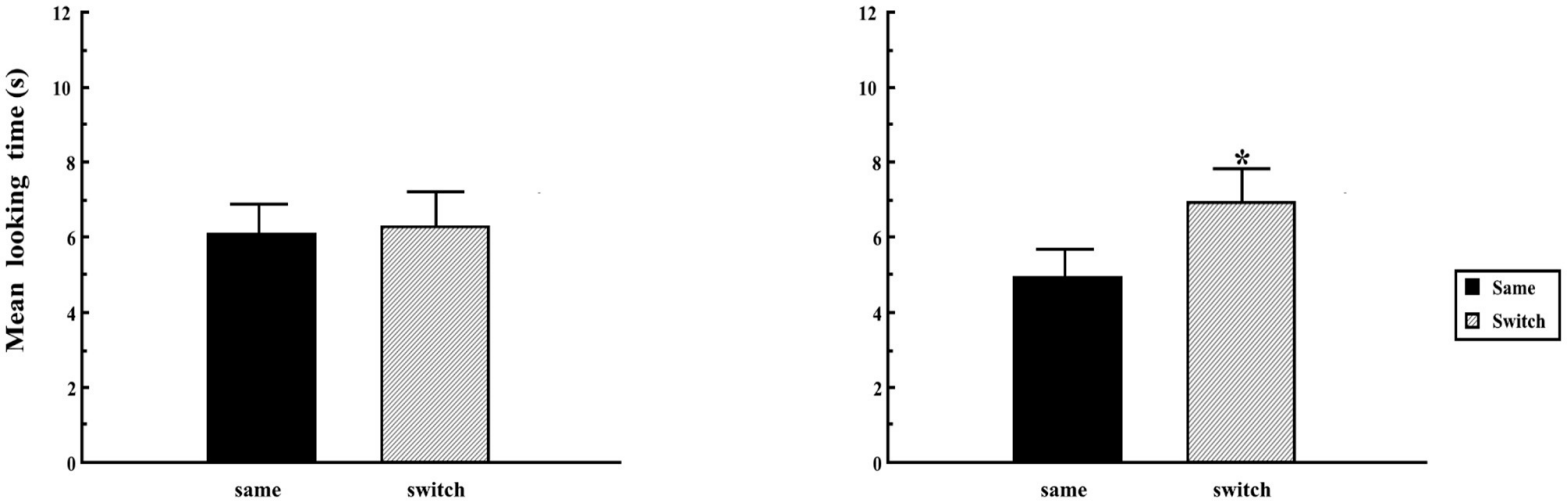
Experimental results from Rost and McMurray (2009): 14 month-old infants do not have significantly longer looking time to the labeled object on switch trials after exposure to a single speaker **(left)** but they do after exposure to multiple speakers **(right)**. The asterisk indicates a statistically significant increase in looking on the switch trials compared to the same trials.

We focus on one variation on the Switch task experiment by Rost and McMurray (2009), which showed that exposure to multiple speakers during habituation helps support 14-month-old infants' success on the Switch task. Rost and McMurray (2009) trained infants on two lexical neighbors (“buk,” “puk”) in a Switch task, with stimuli recorded either from a single speaker, or from a total of 18 different speakers. Unlike the infants who heard exemplars recorded in a single voice, infants in the condition with multiple speakers successfully discriminated lexical neighbors on the switch trials. Figure 4 shows that the difference in looking time between same and switch trials is enhanced in the condition with multiple speakers.

The authors attributed the infants' success in the multiple-speaker condition to a greater availability of useful phonetic variation in the input. Apfelbaum and McMurray (2011) subsequently modeled these results using associative learning principles. Instead of the infants attending to the acoustic cues which indicate the speech contrast being tested, their account predicts that infants may be attending in their perceptual learning primarily to acoustic cues characteristic of a particular speaker—in their model, characteristics of the speaker's voice are mistakenly associated with the object. Attending to speaker-specific cues causes the model to fail to register the phonological contrast between two labels simply because they were spoken by the same person. In the multiple speaker condition, where cues to speaker identity are different with each observed token, it is not possible for the learner to make this mistake.

This explanation for differences between the single speaker and multiple speaker Switch task conditions relies on the supposition that infants are demonstrating immaturity in their knowledge about which phonetic cues are relevant for distinguishing object labels. Rost and McMurray (2009) and Apfelbaum and McMurray (2011) both argued that exposure to a more diverse data set better facilitated the categorical learning by clarifying which cues were relevant for distinguishing the label.

However, although maturation in general knowledge of phonetic variation is one possible factor driving the Switch task results, there is already evidence that sociophonetic perception may play a role in guiding infant attention to phonological contrasts: 14 month old performance on the Switch task can be disrupted by non-phonological sociophonetic cues to gender (Quam et al., 2017). Moreover, the presentation of multiple speech sources may license a social inference that agreement among the speakers is indicative of reliability (Fennell and Waxman, 2010). We formalize this idea of social inference and show that our model predicts infants' behavior in Rost and McMurray (2009). Moreover, contrary to previous models, it predicts that infants will exhibit this behavior even if they can categorize speech sounds and identify the referent of the speech act in an adult-like way.

### 5.2. Model

We use the model from Shafto et al. (2012) of reasoning about categories and speakers of unknown reliability to simulate two experiments from Rost and McMurray (2009), contrasting the behavior of infants habituated to exemplars which were produced by either a single speaker, or by multiple speakers. We model the seven unique speakers that infants heard; in our simulation, we treat the learner as having received one data point from each speaker. In the previous simulation we presented, infants were able to directly observe what groups informants belonged to, and use their knowledge of familiar labels to infer informant quality, however, the experiments from Rost and McMurray (2009) test infants on their ability to learn novel words. We therefore assume that the infant's task includes inferring, rather than observing, the informants' group membership, and learning the novel label. Specifically, they observe some data *D* and infer the correct category *C* under uncertainty about speaker knowledgeability *K*. In the single speaker condition, all observations are attributed to one source, whereas in the multiple speaker condition, each observation may be attributed to a different source.

The simulations presented here use a hypothesis space of three possible labels (puk, buk, and duk) for illustrative purposes, but the qualitative results are not driven by the number of categories. We assume a uniform prior on the label *C*. We also assume that the prior probability of any given informant being knowledgeable is 0.5; this encodes the type of maximum uncertainty that might come from integrating over all possible groups.

The participants in Experiment 1 from Rost and McMurray (2009) heard seven consecutive instances of the same exemplar. We simulate the beliefs of the infant at the end of this habituation period by using our model to calculate the joint posterior probability of the category and the speaker's knowledgeability after seven instances of the same unambiguous token. We then compute the extent to which the model believes that the label seen during habituation is the correct label by computing the model's marginal posterior distribution over labels.

We model the infant's looking time as the result of a joint inference on (*C, K*) for a sequence of data points $\vec{D}$. We predict that looking time will correlate with certainty about the speaker's reliability. In this condition, all the data points are associated with a single belief about the knowledgeability of the speaker. The joint posterior probability of the category and speaker's knowledgeability is given by Equation (5).

(5) $$\begin{array}{r} P\left(C,K|\vec{D}\right)=\frac{P\left(\vec{D}|C,K\right)P\left(C,K\right)}{\underset{C^{\prime },K^{\prime }}{\sum }P\left(\vec{D}|C^{\prime },K^{\prime }\right)P\left(C^{\prime },K^{\prime }\right)} \end{array}$$

For the condition where the infant hears labels from multiple speakers, we model this as a joint inference on $\left(C,\vec{K}\right)$ for a sequence of data points $\vec{D}$. In this condition, each data point is the contribution of a distinct speaker, and as such is associated with a unique belief about knowledgeability specific to this speaker. In other words, for a set of data with *m* elements, the listener must now infer a sequence $\vec{K}$ with length *m*.

(6) $$\begin{array}{r} P\left(C,\vec{K}|\vec{D}\right)=\frac{P\left(C,\vec{K}\right)P\left(\vec{D}|C,\vec{K}\right)}{\underset{C^{\prime },K^{\prime }}{\sum }P\left(C^{\prime },\vec{K^{\prime }}\right)P\left(\vec{D}|C^{\prime },{\vec{K}}^{\prime }\right)} \end{array}$$

Table 2 illustrates how the size of the hypothesis space grows when observing three speakers. With the evaluation of additional speakers, it would continue to grow exponentially. This makes the posterior distribution too complex to calculate analytically, so we instead use Gibbs sampling, a Markov chain Monte Carlo method that allows us to perform approximate inference (Geman and Geman, 1984). Given the conditional distributions, $P\left(C|\vec{K},\vec{D}\right)$ and $P\left(\vec{K}|C,\vec{D}\right)$, the Gibbs sampler iteratively samples from these, using the new value obtained at each step to sample from the other conditional distribution. This iterative sampling process converges to approximate the joint distribution described in Equation (6). For a more detailed description, see Appendix 2.

**Table 2:** Hypothesis space given a scenario where the object label (*C*) is known, but the knowledgeability of three informants (*K*_1_, *K*_2_, *K*_3_) is unknown.

**Category and first speaker** **(*C, K*_1_)**	**Second speaker** **(*K*_2_)**	**Third speaker** **(*K*_3_)**
*C*= buk, *K*_1_ = 0	*K*_2_ = 0	*K*_3_ = 0
		*K*_3_ = 1
	*K*_2_ = 1	*K*_3_ = 0
		*K*_3_ = 1
*C*= buk, *K*_1_ = 1	*K*_2_ = 0	*K*_3_ = 0
		*K*_3_ = 1
	*K*_2_ = 1	*K*_3_ = 0
		*K*_3_ = 1

### 5.3. Results

In modeling looking times in the Switch task, we assume that increased certainty about the label, *C*, is expected to correlate with increased looking to the target image on switch trials, as a result of infants being more surprised at the novel label. By contrast, infants who are unsure of the label should demonstrate lower looking times, because their existing uncertainty makes a new label less surprising.

Table 3 shows the posterior probability the model assigned to each label after familiarization, across the two conditions. The table shows the predicted probability of each label, or category, *C*, being the correct name for the object, given an observation of data, *D*. In this case, the infant has observed seven tokens of the label “buk” associated with the object during habituation. The model predicts that infants will be much more confident of the object label after hearing multiple speakers agree. This occurs because agreement among speakers increases the model's belief that not all the speakers are unknowledgeable. Since unknowledgeable speakers do not always produce the correct label, this makes it more likely that speakers in agreement are knowledgeable, and have supplied the correct label. These results demonstrate that infant behavior can be predicted by the same model of reasoning about epistemic trust that described the behavior of 4 and 5 year old children.

**Table 3:** Posterior probability of the label after seven observations of “buk.”

	**Single speaker**	**Multiple speaker**
**Label**	**P(*C*∣*D*)**	**P(*C*∣*D*)**
C = “buk”	0.6667	0.9992
C = “puk”	0.1667	0.0004
C = “duk”	0.1667	0.0004

### 5.4. Discussion

The simulation in section 4 applied a computational model to simulate infant behavior which is well-accepted as attributable to infants' evaluation of informant quality. The simulation of infant perception of labels in Rost and McMurray (2009) demonstrates that experiments in the literature that haven't previously been considered in the context of social inference may nevertheless be explained that way. We demonstrate that the pattern of results can be interpreted as evidence of social inference in word learning. Rather than changes in the infant's capacity for phonetic learning, social development may be the best explanation for observed behavioral changes, with infants making increasingly sophisticated judgements about group membership which selectively facilitate the learning of fine phonetic detail only in appropriate social scenarios. We argue that the cognitive development which underpins infant progress on the Switch task may be governed by changes in the meta-cognitive function of epistemic trust. Rather than simply reflecting more sophisticated phonetic perception, improvement on the Switch task at 18 months may reflect infants' developing strategies for determining informant group membership and reliability. Fine grained attention to contrastive phonetic cues may be gated by the infant's perception of an informant as giving reliable information about the phonetic characteristics of a particular language variety. Accordingly, we can explain the infant's ability to learn more precise phonetic detail by attributing to the infant (1) knowledge of an informant's group membership, and (2) the expectation that sources affiliated with reliable groups provide superior testimony. In the presence of apparently less reliable sources, we still predict that infants will learn, but that rather than acquiring fine phonetic detail, they will instead learn broader phonetic contrasts. In contexts where the presence of more reliable sources can be more confidently inferred, we predict that learners will selectively attend to phonetic detail provided by individual informants judged to be knowledgeable. While the majority of Switch task studies have attempted to measure infant recognition of words by assuming that the representational structures at issue did not reflect any meaningful variation in encoding of details about informants or their reliability, we argue that their results nevertheless also support our conclusions.

In what follows, we discuss two main alternative explanations that are often given to account for findings in the Switch task literature, and discuss their compatibility with our account. The first is that infants who fail on the Switch task do so due to a resource limitation. The second is that infants who fail on the Switch task do so due to the ambiguity or absence of referential cues. We argue that the source selection hypothesis is a parsimonious explanation which effectively unites both accounts.

#### 5.4.1. The Cognitive Load Account

Associating an object with a label also requires the coordination of other cognitive processes, including attention, segmentation, and inference about the speaker's referential intent. The failure to demonstrate phonemic discrimination on the Switch task has sometimes been attributed to a resource limitation (Stager and Werker, 1997; Pater et al., 2004). The source evaluation account is compatible with these accounts, however it further provides a specific falsifiable prediction regarding the difficulty of the task: word learning is most likely to be evident under task conditions which facilitate inferences that the informant is a member of a trusted social group. Developmental changes on the Switch task may be best explained with reference to changing strategies for evaluating informants.

The cognitive load hypothesis is supported by infant improvement in performance on variations on the Switch task which are designed to be easier. Fourteen-month old infants perform above chance on a simpler Switch task administered with a preferential looking paradigm, consistent with the hypothesis that declines in performance observed under other conditions are the result of task difficulties (Yoshida et al., 2009). This slight preference is predicted by the model given in Apfelbaum and McMurray (2011), and under the assumption that the experimental participant assigns the event that the speaker in the habituation phase is knowledgeable a non-zero probability, our model predicts more looking to the labeled object during a preferential looking test as well.

In tasks involving familiar words and objects, 14-month-olds demonstrate increased sensitivity to phonetic detail (Swingley and Aslin, 2002; Fennell and Werker, 2003, 2004; Fennell, 2012). Supposing at least part of infants' difficulty in succeeding at the Switch task with minimally different labels is attributable to the increased task requirements of the audio-visual associative learning required to respond to novel words, then the presentation of familiar stimuli should alleviate that difficulty. In effect, the participants' prior knowledge may facilitate the task.

In our model, we can simulate this contrast by increasing θ_*g*_, the characteristic group knowledgeability. The parameter *K* in our model predicts the likelihood of an informant both correctly identifying and labeling the referent, which, whether familiar or novel, is known to the experimental participant. Assuming that the infant believes that a familiar object is more likely to be known to their interlocutor, or that the object is simply more salient (and thus more likely to be known), an increase in *P*(*K*|*G*) simulates the effect of familiar stimuli. Rather than the familiarity of the lexical items facilitating lexical processing, it may facilitate epistemic trust in the informant, indirectly resulting in greater phonetic sensitivity.

#### 5.4.2. The Referential Ambiguity Account

Performance on the Switch task improves when the novel word is embedded in an overtly referential phrase (i.e., “look at the *blick*”) (Fennell and Waxman, 2010) or when the training phase contains familiar named objects (Fennell et al., 2007). However, when familiar objects in habituation are paired with exclamations (e.g., “Wow!” or “Whee!”), no improvement is observed (Namy and Waxman, 2000). These results have been interpreted as support for the hypothesis that 14-month-old infants' failure on the Switch task is a consequence of referential ambiguity. Cues which make the stimulus presentation more clearly a referential act increase the likelihood that infants demonstrably create a mapping between the word and object using fine phonetic detail.

Other studies demonstrate that the infants are more likely to succeed on the Switch task when additional referential cues are present. For example, 14-month olds look longer to switch trials after being exposed to pre-test trials showing the speaker labeling familiar objects (Fennell and Waxman, 2010). Fennell and Waxman (2010) interpreted these results to indicate that the infants were assisted in making inferences about the referential intentions of the speakers. In other words, strong referential cues helped the infants infer that the provided label was intended by the speaker to correspond with the object.

Our hypothesis is consistent with this one, but makes an important distinction in scope. Because Fennell and Waxman (2010) discuss infant inference about the referential nature of observed speech provided by a single individual, these data cannot distinguish between hypotheses which rely on characterizing the infant's perception of the specific informant's intentions and the infant's perception of the label when used by other informants. It is possible that the participants interpreted pre-test trials displaying accurate labeling behavior as evidence that the speaker is not only intentionally engaging in referential acts, but is doing so credibly as a member of the infant's social and linguistic in-group. The subsequent improvement in performance for these infants may demonstrate that they formed a belief that beyond their intent to refer, the speaker is epistemically a source of accurate linguistic data. Rather than simply inferring whether the speaker intends to label the object, the participant may also be concerned with whether the label is accurate and likely to be used by other speakers of their language. The latter inference is captured by our model.

The task is designed to make the labeled object salient to the experimental participant, so if we assume, as we have before, that the infants do know which object is being referred to, the inclusion of additional cues that the speech act references this object may again be encoded as an increase in the parameter θ_*g*_. Rather than simply tracking the speech acts themselves, a listener who is also sensitive to source may interpret additional referential cues as a reflection on the individual knowledgeability or group membership of the linguistic informant. We expect any stimuli which bias the infant to believe the informant is more likely to select both the correct referent and label will also result in them being more surprised on switch trials, consequently improving performance on the task.

Inferences about the quality of the informant may also account for evidence from Galle et al. (2015) that increased acoustic variability helps infants' performance on the Switch task even when the increased variability is provided by a single talker. The variability of stimuli in Galle et al.'s experiment were designed to be naturalistic, varying across several speaking styles. The presented acoustic variation is therefore likely similar to the sort infants are accustomed to regularly encountering from reliable linguistic informants in their environment, potentially increasing infants' prior beliefs about *K*.

Increased visual variability in the presented objects, however, does not appear to improve infant performance (Höhle et al., 2020). Meta-analysis of Switch task experiments reveals that language-typical words are easier for infants to learn, and show a consistent advantage for bilingual infants over monolingual infants (Tsui et al., 2019). These findings are consistent with the hypothesis that performance on this task reflects infant social evaluation of informants for their knowledgeability about specific dialects.

## 6. General Discussion

In this paper, we have analyzed two previous infant word learning experiments and have shown that their results can be predicted by a model which incorporates infants' beliefs about the relative value of informants. We extended a model from Shafto et al. (2012), created to describe word learning behavior in 5 year old children, which posits that learners recruit perception of the probability that talkers will correctly label objects. The word learning task in this model is described as relying upon inferences about both the label and the quality of the source. We then showed that this probabilistic model effectively simulates two experiments from the literature on infant perception of labeling: Koenig and Echols (2003), which investigates the looking patterns of infants to objects given exposure to familiar labels, and Rost and McMurray (2009), which investigates the looking patterns of infants to objects following habituation to a novel label.

The explanation provided for infants' behavior by our first simulation is similar to the original interpretation given by the authors of the study, but our simulation embeds that explanation within a formal model of socially informed word learning. Our extension of the model from Shafto et al. (2012) describes how learners might generalize beliefs about epistemic trust across groups of talkers, allowing the characteristics of a talker's group to constrain listeners' reasoning about the meaning of specific utterances.

In our second simulation, we have further demonstrated that infants recruiting these beliefs about characteristically knowledgeable groups may also explain their behavior in contexts where informant knowledgeability is unknown. Using metalinguistic knowledge regarding which combinations of speech varieties are likely being deployed by the multiple talkers, the learner can use the collective testimony of talkers who are individually of unknown quality as evidence to reason about the current talker's knowledgeability. This explanation differs substantially from previous accounts: rather than immature phonetic representations, our account attributes infants' trouble in the Switch Task to their developing sociolinguistic competence. Word learning settings may be more difficult than simple phonetic discrimination precisely because infants are performing the more complex task of inferring speaker trustworthiness. Both simulations demonstrate that infants' looking behavior when hearing words may be explained with reference to social beliefs about the likelihood of agreement among talkers.

While the model set forth in this paper has not previously been used to describe infant language acquisition, we have shown that it provides a potential explanation for effects already established in the literature. Our simulations demonstrate that language learners may well be recruiting processes of epistemic trust to guide lexical acquisition much earlier than previously suggested.

Our results point to a new theoretical framework in which the object of lexical learning is not simply the acquisition of lexical forms, but rather, a metalinguistic framework for reconciling competing hypotheses about the lexical identities of individual speech tokens. Under this account, the learner's attention is distributed according to beliefs about the social and dialect group membership of the source, and the listener's perception of their own possible accuracy in identifying informants who are knowledgeable about their target dialect. The less likely a learner believes they are to be able to correctly predict whether a source is knowledgeable, the more certain they may then be that attending to this source will be surprising and informative about the speaker's knowledgeability. On the other hand, the more certain a learner can expect to be about the knowledgeability of an individual talker *before observing their speech*, the more likely they will be to judge that talker's testimony uninformative. Perfectly knowledgeable informants are predicted to produce useful, novel information about knowledgeability at lower rates than imperfect informants whose behavior is overall more unpredictable. The labels which will be most informative about the distribution of knowledgeability will therefore be incorrect labels produced by informants who are expected to be knowledgeable.

Therefore, we may describe language learners as fruitfully relying on differences in the relative perception of uncertainty to discriminate between linguistic informants. Differences in the breadth or specificity of listener social beliefs about contrasts between kinds of speakers may also predict differences in task complexity, both within groups and at the level of individual differences. Infants from different backgrounds may take different approaches to the same apparent task. Learners who have beliefs which cause them to more confidently discriminate among informants are predicted to selectively respond to informants they believe knowledgeable, displaying increased precision in their phonetic perception. Contrastively, we predict that learners with more uncertainty about the distribution of knowledgeable informants will display both weaker informant preferences and coarser phonetic perception. We predict that improvement on the Switch task with age can be explained by older infants requiring fewer contextual cues to resolve ambiguity between possible interpretations.

In what follows, we contextualize our model relative to the larger literature surrounding children's sociolinguistic competence during language acquisition and lay out our broader proposal for conceptualizing the problem of word learning. We then sketch fruitful directions for future work.

### 6.1. Evidence of Infant Sociolinguistic and Metalinguistic Knowledge

Our findings show that the receptiveness of preverbal infants to both familiar and novel lexical items can be described as the result of infant beliefs regarding the informant's membership in abstract social groups. Although our framing of the word learning problem as also involving social inference is a novel perspective with respect to models of word learning, it is consistent with a large body of literature showing that even very young children have substantial sociolinguistic competence. For example, 12 month old have been shown to use talker specific voice characteristics to learn talker-dependent linguistic structure, successfully generalizing grammatical rules learned from a single talker to sentences from novel talkers (Gonzales et al., 2018).

Language variation is correlated with social differences from an early age. Along with linguistic knowledge, developing language users also acquire social identities which influence their linguistic behavior. For example, gendered differences in speech are often associated with biological distinctions caused by sexual dimorphism, such as vocal tract length. However, gendered differences have been found to emerge in the speech of children before their physical development diverges. Perry et al. (2001) found that adults rated the speech of children aged 4–8 years as having distinctive genders, despite the fact that vocal tracts of boys and girls at this stage of development are structurally indistinguishable. Likewise, differences in gendered speech are not consistent across languages, as would be expected if they were solely predicted by physical characteristics (Cherng et al., 2002). Even within isolated and ethnically homogeneous populations, children inevitably acquire and deploy culturally specific metalinguistic and sociolinguistic knowledge.

The literature also provides evidence for the integration of linguistic and non-linguistic talker perception processes in infants. Infants represent differences between groups of talkers, and use this information to guide both their linguistic and non-linguistic social preferences, preferring to socially engage with talkers who use familiar rather than unfamiliar languages (Kinzler et al., 2007). In the second year of life, children show some evidence of being sensitive to cues that the speaker is uncertain about referring to an object, preferentially learning labels in conditions where the label is spoken with confidence (Brosseau-Liard and Poulin-Dubois, 2014).

By 5 years of age, children preferentially select same-race social partners, but show even stronger preferences for talkers with familiar over foreign speech accents (Kinzler et al., 2009). “In-group identity” is commonly accepted to emerge early, arising from processes of self-categorization (Spears, 2020). However, there has been very little previous work investigating how this kind of categorical social perception influences lexical learning. Infants are known to rely on knowledge of the in-group to guide their social learning, but we can expect social knowledge to reciprocally affect their language acquisition. Linguistic and non-linguistic notions of in-group status appear to be intrinsically connected.

If infants' speech expectations are affected by perception of speaker affiliations, such as speech accent and appearance, we could expect infants to interpret phonetic information differently depending on the visual presentation of the talker. Weatherhead and White (2018) provide some preliminary experimental evidence to support this claim. In this study, 16 month old infants were exposed to familiar words either in a familiar or unfamiliar accent. The 16 month old infants possessed some expectations about accent and race, namely that familiar-race speakers are likely to pronounce words in familiar ways, while unfamiliar-race speakers are not. This evidence suggests that language learners' mental representations are associated with beliefs about identity, and that a perception of shared identity may make speech easier to process.

Corriveau et al. (2016) exposed children of backgrounds with varying socio-economic status (SES) to informants who either used passive or active voice constructions to describe images. They found that children from high SES backgrounds show a preference to learn novel words from informants who used the more complex syntax, while children from lower SES backgrounds preferred the informants who used simpler sentence structure. The finding suggests that despite both groups of children demonstrating understanding of the more complex syntactic form, that the relative amount of experience with informants who use each form predicted their selective trust in novel informants exhibiting the same behavior.

It is clear from this literature that perceptions of social and linguistic similarity are correlated. The present model demonstrates an explanation for why. Both may be interpreted a product of learners seeking informants who are representative of the epistemically preferable in-group, having both linguistic and non-linguistic attributes. The relationship between perception of social and linguistic groupings is bidirectional; an informant's linguistic attributes influence the interpretation of their non-linguistic behavior, and an informant's non-linguistic attributes likewise influence the interpretation of their linguistic behavior. It is not possible to address bias in the use or study of language without acknowledging how linguistic and non-linguistic cognitive processes are necessarily intertwined.

### 6.2. Reconceptualizing the Word Learning Problem

The literature reviewed in the previous section on the interaction between social groups and language, together with our modeling results showing that social inference likely plays a role in word learning, suggests that we need to reconceptualize the word learning problem. Instead of framing the word learning problem as proceeding in an asocial way, we need to view it as requiring learners to compare different talkers' social value as informants for language acquisition. The abstraction of a homogeneous speech community effectively denies the well documented ability of children to perceive contrastive social value between multiple alternative linguistic codes, and differences in their effect when employed by distinctive types of talkers. Incorporating non-linguistic talker perception and epistemic trust into a word learning model allows us to begin remedying several consequences of the asocial conceptualization of word learning.

Firstly, the present model permits the learner to perceive multiple varieties of the same language. In order to acquire a specific variety, the learner must be able to differentiate talkers using the dialect which they aim to acquire from talkers using different varieties—that is, they must be able to perceive speakers as more or less representative talkers of the target variety. Rather than generalizing observed variations in speech and communication patterns to all talkers, language learners' generalizations intrinsically describe contrasting subsets of talkers, each speaking a different variety of the same language. Our model presumes a learner who may interpret non-linguistic features as signals of linguistic utility through associative learning, and use these to identify subsets of talkers who may be more or less useful for acquiring their target variety.

However, whereas in our model, learners filtered out data from the non-target variety, this is unlikely to be accurate: developing a preference to imitate the acoustic patterns of, for example, either male or female talkers does not prevent children from acquiring linguistic units from the dispreferred category of talkers; the value of a particular informant may therefore eventually need to be modeled with a gradient measure of utility relative to a desired subset of talkers, rather than a categorical distinction between knowledgeable and unknowledgeable talkers. In this way, learners acquiring a target dialect would be able to draw on data even from informants who don't speak their target dialect, but who do speak the same language; but they would still treat data from same-dialect vs. different-dialect informants in fundamentally different ways.

Positing that there is one language variety which is considered more representative of knowledgeability than others predicts that a speaker who recognizes multiple dialects of a language will nonetheless favor one dialect over others. Such a model can potentially account for how learners develop principled beliefs about the form and content of speech from same race and other race informants, as well as the role of vernacular and standard dialectal items and structures within a given community of practice. Early in development listeners might to respond differently to dialects not just as a function of exposure but of attitudes toward the speech affected by the beliefs about the quality of that exposure.

Data from knowledgeable speakers will aid in the prediction of both what referential content other knowledgeable speakers provide and what referential content they will not. Conversely, data from unknowledgeable speakers is not helpful in predicting the referential content of labels from either knowledgeable or other unknowledgeable speakers. In effect, knowledgeable speakers are not only expected to produce reliable speech, they are also expected to produce speech which can be **reliably recognized as reliable**, inducing in the listener not only a greater degree of confidence in their interpretation of the speech, but also a greater degree of confidence in the metalinguistic framework which produces that interpretation.

Previous computational models of language acquisition have relied on the abstraction that infants wholly dissociate their perception of the speech from that of the speaker, and therefore these models cannot predict any of the known effects of source evaluation on acquisition. We have shown that with incremental departures from the abstraction of an asocial learner expecting a monodialectal input, we are able to better capture patterns of infant behavior on tests of word knowledge. The perception of affiliative cues signaling informant group membership is therefore expected to have significant effects on language outcomes. Rather than excluding linguistic and non-linguistic affiliative judgements of language users from our understanding of the word learning problem, we instead argue that we should define learners as necessarily recruiting these non-linguistic judgements to validate their solutions to the language learning problem.

### 6.3. Investigating Infant Knowledge About Informants

Both of the simulations we have presented suggest that preverbal infants are epistemically evaluating sources of linguistic information. In effect, infants who are failing to attend to informants and detail in their speech may be demonstrating an expectation that the source and/or their data are not trustworthy. Experiments which control for infants' perception of source reliability are needed to provide more explicit support for this interpretation of the literature. To explore the link between processes of epistemic trust, social inference, and language acquisition, several directions are fruitful avenues for future research.

A crucial prediction of our theory is that in acquiring a specific speech variety, not all sources will be equally useful to a language learner. If the child is rationally interpreting evidence of label variation in a social setting, we should expect their attention to be distributed in accordance with their beliefs about the usefulness of speakers and kinds of speakers. There are a number of ways one could test this, including within existing paradigms such as the Switch task.

For example, suppose a Switch task preceded by a habituation featuring labeling from two speakers. The source-tracking hypothesis predicts that whether listeners demonstrate sensitivity to a phonetic contrast will be partially predicted by their belief that the speaker is knowledgeable. Supposing one of the speakers heard in pre-test is more reliable at labeling familiar objects, infants who hear this speaker's voice on test trials should be more likely to attend to switch trials than infants who hear the less reliable speaker's voice at test. Likewise, the use of a pre-test demonstration where the speaker is shown to be more or less reliable using non-linguistic cues (such as indicating with gaze where an object will appear) may diminish the beneficial effect of naming familiar objects pre-test. If infants are attending to the reliability of the speaker, then demonstrations that they are unknowledgeable in other ways may cause the infant to disprefer attending to that informant's phonetic variation.

More broadly, under the source selection account, preferences for informants which are formed early on may have far reaching effects, shaping the development of the lexicon for years afterwards. To explore this hypothesis, it is necessary to conduct a systematic comparison of infant performance after exposure to different amounts of testimony from differing numbers of informants. It is also necessary to determine how allocation of epistemic trust may vary between populations. Children from different cultural backgrounds and learning in different modalities are expected to eventually acquire distinct strategies for determining the reliability of an informant. Therefore, before we may tease apart the effects of exposure and epistemic trust on word learning, we must understand normal variation in its application. The present work suggests a new research program uniting studies of developmental social psychology with psycholinguistic processing, to discover how variation in phonetic representations are affected by the perception of identity, including attributes such as authority, gender, and race.

We have shown that uniting accounts of selective trust with language learning has the potential to deepen our understanding of many areas of linguistic study. A research program in early sociophonetic learning has the potential to increase understanding of variation in language outcomes owing to differences in cultural background, identity, and disordered language skills. In applied linguistics, it may assist in understanding the etiology of academic achievement gaps, or functional differences between typically developing and developmentally disabled language users.

## Data Availability Statement

The original contributions presented in the study are included in the article/supplementary material, further inquiries can be directed to the corresponding author/s.

## Author Contributions

AT, NF, and WI designed the research, developed the model, and edited it. AT implemented the model, conducted the simulations, and analyzed the results. AT wrote the initial draft of the manuscript. All authors contributed to the article and approved the submitted version.

## Conflict of Interest

The authors declare that the research was conducted in the absence of any commercial or financial relationships that could be construed as a potential conflict of interest.

## 1. Appendix 1: Modeling Listening to Familiar Words

In our first simulation we model data from Koenig and Echols (2003). We use Kullback-Leibler divergence, which describes the relative entropy of a distribution compared to a reference distribution, to measure the average number of bits learned about the speaker's knowledgeability after hearing them label an object. Specifically, we measure the difference between the posterior distribution over knowledgeability to the prior distribution,

(A1) $$\begin{array}{l} D_{KL}\left(P\left(K|D,G,C\right)||P\left(K|G,C\right)\right)\\ \;=\underset{K}{\sum }P\left(K|D,G,C\right)\log\frac{P\left(K|D,G,C\right)}{P\left(K|G,C\right)} \end{array}$$

We expand the posterior distribution *P*(*K*|*D, G, C*) using Bayes' rule,

(A2) $$\begin{array}{r} P\left(K|D,G,C\right)=\frac{P\left(D|K,C\right)P\left(K|G\right)}{\underset{K^{\prime }}{\sum }P\left(D|K^{\prime },C\right)P\left(K^{\prime }|G\right)} \end{array}$$

where *K* is independent of *C*, and *D* is independent of *G* when conditioned on *K*. Substituting this expression into Equation (A1) and canceling terms yields

(A3) $$\begin{array}{r} \underset{K}{\sum }\frac{P\left(D|K,G,C\right)P\left(K|G,C\right)}{\underset{K^{\prime }}{\sum }P\left(D|K^{\prime },G,C\right)P\left(K^{\prime }|G,C\right)}\log\frac{P\left(D|K,G,C\right)}{\underset{K^{\prime }}{\sum }P\left(D|K^{\prime },G,C\right)P\left(K^{\prime }|G,C\right)} \end{array}$$

The prior on knowledgeability, *P*(*K*|*G*), is given in Equation (1), and the likelihood term, *P*(*D*|*K, C*), is given in Equation (3).

## 2. Appendix 2: Modeling Listening to Familiar Words

In our second simulation we model data from Rost and McMurray (2009). Infants in the single speaker condition heard several datapoints from the same speaker. We assume they jointly inferred the label *C* for the object and the knowledgeability *K* of the speaker using Bayes' rule,

(A4) $$\begin{array}{r} P\left(C,K|\vec{D}\right)=\frac{P\left(\vec{D}|C,K\right)P\left(C\right)P\left(K\right)}{\underset{K^{\prime },C^{\prime }}{\sum }P\left(\vec{D}|C^{\prime },K^{\prime }\right)P\left(C^{\prime }\right)P\left(K^{\prime }\right)} \end{array}$$

where we assume uniform priors over *C* and *K*, and the likelihood $P\left(\vec{D}|C,K\right)$ is the product of the likelihoods of the data points, ∏_*j*_*P*(*D*_*j*_|*C, K*). This likelihood function is given in Equation (3).

In the multiple speaker condition, infants heard datapoints from several speakers. We assume they jointly inferred the label *C* for the object and the knowledgeability *K* of each of the speakers, $P\left(C,\vec{K}|\vec{D}\right)$.

Because the number of hypotheses grows exponentially With the number of speakers, we use Gibbs sampling to estimate this posterior distribution. We initialize *C* by sampling a uniform multinomial distribution and alternate between sampling $\vec{K}$ by sampling each speaker's *K* from its conditional distribution given current beliefs about the true label of the object,

(A5) $$\begin{array}{r} P\left(K_{j}|C,D_{j}\right)=\frac{P\left(D_{j}|C,K_{j}\right)P\left(K_{j}\right)}{\underset{K_{j}^{\prime }}{\sum }P\left(D_{j}|C,K_{j}^{\prime }\right)P\left(K_{j}^{\prime }\right)} \end{array}$$

and resampling *C* from its conditional distribution given current beliefs about the knowledgeability of the speakers,

(A6) $$\begin{array}{r} P\left(C|\vec{K},\vec{D}\right)=\frac{P\left(\vec{D}|C,\vec{K}\right)P\left(C\right)}{\underset{C^{\prime }}{\sum }P\left(\vec{D}|C^{\prime },\vec{K}\right)P\left(C^{\prime }\right)} \end{array}$$

where *j* indexes over data points and each data point is assumed to have been produced by a different speaker. The likelihood term $P\left(\vec{D}|C,\vec{K}\right)$ in Equation (A6) is the product of the likelihood terms for each data point, ∏_*j*_*P*(*D*_*j*_|*C, K*_*j*_). This likelihood, also used in Equation (A5), is given in Equation (3). We again assume uniform priors over *C* and *K*. The Gibbs sampler was run for 1,000 iterations and analysis was done on every third sample after a burn-in period of 100 iterations.
